# Immunobiology of Atherosclerosis: A Complex Net of Interactions

**DOI:** 10.3390/ijms20215293

**Published:** 2019-10-24

**Authors:** Beatriz Herrero-Fernandez, Raquel Gomez-Bris, Beatriz Somovilla-Crespo, Jose Maria Gonzalez-Granado

**Affiliations:** 1 LamImSys Lab. Instituto de Investigación Hospital 12 de Octubre (imas12), 28041 Madrid, Spain; beatriz.herrero@uam.es (B.H.-F.); rgomez.imas12@correo.h12o.es (R.G.-B.);; 2 Departamento de Fisiología, Facultad de Medicina, Universidad Autónoma de Madrid (UAM), 28029 Madrid, Spain; 3 Centro Nacional de Investigaciones Cardiovasculares Carlos III (CNIC), 28029 Madrid, Spain; 4 CIBER de Enfermedades Cardiovasculares, 28029 Madrid, Spain

**Keywords:** atherosclerosis, monocyte, foam cell, macrophage, monocyte-derived dendritic cell, T-cell, B-cell, conventional dendritic cell, plasmacytoid dendritic cell, regulatory dendritic cell

## Abstract

Cardiovascular disease is the leading cause of mortality worldwide, and atherosclerosis the principal factor underlying cardiovascular events. Atherosclerosis is a chronic inflammatory disease characterized by endothelial dysfunction, intimal lipid deposition, smooth muscle cell proliferation, cell apoptosis and necrosis, and local and systemic inflammation, involving key contributions to from innate and adaptive immunity. The balance between proatherogenic inflammatory and atheroprotective anti-inflammatory responses is modulated by a complex network of interactions among vascular components and immune cells, including monocytes, macrophages, dendritic cells, and T, B, and foam cells; these interactions modulate the further progression and stability of the atherosclerotic lesion. In this review, we take a global perspective on existing knowledge about the pathogenesis of immune responses in the atherosclerotic microenvironment and the interplay between the major innate and adaptive immune factors in atherosclerosis. Studies such as this are the basis for the development of new therapies against atherosclerosis.

## 1. Immune system 

**Figure 1:**
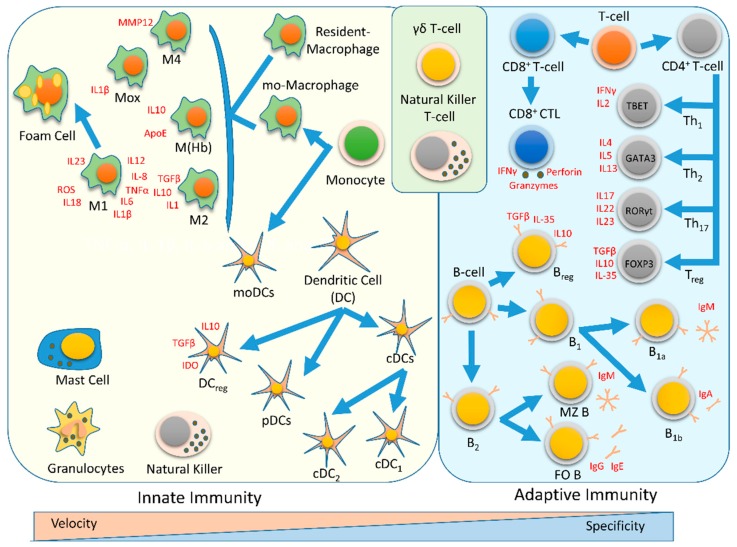
The innate and adaptive immune system in atherosclerosis. The immune system is divided into two main branches: innate and adaptive immunity. Innate immunity is mediated by monocytes, macrophages, dendritic cells, granulocytes, mast cells, and natural killer cells, and is characterized by the capacity of these cells to produce a rapid and nonspecific response as a first line of defense. Innate immune cells mediate host defense responses and inflammation by releasing cytokines and chemokines, activating the complement cascade and phagocytosis, and activating the adaptive immune response via antigen presentation. The adaptive immune response occurs later and depends on the presentation of antigens by antigen presenting cells (APCs) and the cytokine milieu generated by the innate response. The adaptive response is specific and relies on CD4^+^ and CD8^+^ T cell activation and antibody production by B cells. Natural killer T (NKT) cells and γδ T cells are cytotoxic T lymphocytes at the interface between innate and adaptive immunity. Abundant evidence points to a role of innate and adaptive immunity in the onset and progression of atherosclerosis. Several immune cell types play a proatherogenic role, including Th1 cells, M1 macrophages, and some B cells. In contrast, Tregs, Bregs, and DCregs have atheroprotective effects. DC, dendritic cell; moDC, monocyte-derived DC; mo-Macrophage, monocyte-derived macrophage; pDC, plasmacytoid DC; cDC, conventional DC; FO B, follicular B cell; MZ B, marginal zone B cell; Breg regulatory B cell; Treg, regulatory T cell.

The immune system is divided into two main branches, the innate and adaptive responses Figure 1. Innate immunity is enacted by cells of the myeloid lineage characterized by their capacity to produce a rapid and nonspecific response as a first line of defense. Innate immune cells can sense pathogen-associated molecular patterns (PAMPs) and damage-associated molecular patterns (DAMPs) through their expression of pattern recognition receptors (PRRs), such as toll-like receptors (TLRs). Innate immune cells mediate host defense responses and inflammation by producing cytokines and chemokines, activating the complement cascade and phagocytosis, or presenting antigens to activate the adaptive immune response. Prominent cells of the innate immune system include neutrophils, macrophages, and dendritic cells (DCs). The adaptive response occurs later and depends on the presentation of antigens by antigen presenting cells (APCs) and the cytokine milieu generated by the innate response. The adaptive response is specific and relies on CD4^+^ and CD8^+^ T cell activation and the production of antibodies by B cells. Natural killer T cells (NKT cells) and γδ T cells are cytotoxic T lymphocytes at the interface between innate and adaptive immunity. Abundant evidence indicates that innate and adaptive immunity both play important roles in the onset and progression of atherosclerosis [1,2,3]. For example, *Csf1*^−/−^ mice, which lack macrophage colony stimulating factor (M-CSF, also known as CSF1) are less prone to developing atherosclerosis [4]. Moreover, mice lacking B and T cells (*Rag1*^−/−^ or *Rag2*^−/−^ mice lacking recombination-activating genes 1 or 2 or mice carrying the severe combined immunodeficiency (SCID) mutation) are resistant to atherosclerosis in the presence of mild hypercholesterolemia [5,6,7,8,9,10].

## 2. Atherosclerosis’s Epidemiology

Atherosclerosis is the leading cause of coronary artery disease, morbidity, and mortality worldwide [11,12]. Almost all individuals have atherosclerotic plaques [13], and even when lipids have been reduced to nominally safe levels and plaque development has been arrested, there is still a high probability of sudden thrombotic events leading to myocardial infarction or stroke [14]. Atherosclerosis is characterized by systemic inflammation, and high levels of C-reactive protein (CRP), a circulating predictor of inflammation [15], are found in patients, so it has been proposed as a biomarker of atherosclerosis [16]. Atherosclerosis is a silent disease until increased intimal thickening eventually either diminishes or blocks blood flow, inducing ischemia in downstream tissues, or triggering thrombosis after atherosclerotic plaque rupture [17,18], resulting in myocardial infarction (MI) or stroke [19].

## 3. Atherosclerosis’s Pathophysiology

**Figure 2:**
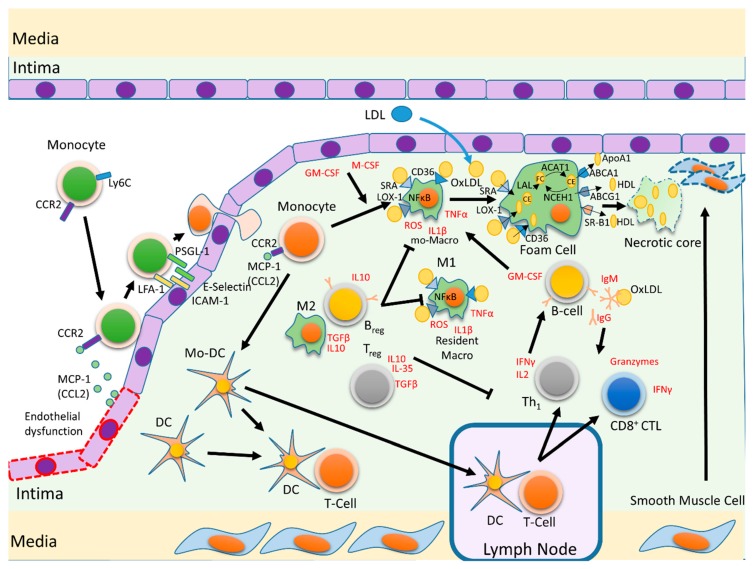
Pathogenesis of atherosclerosis. Atherosclerosis is a chronic inflammatory disease characterized by endothelial dysfunction and accumulation of low-density lipoproteins (LDLs), immune cells, and necrotic debris in the subendothelial space. Endothelial activation triggers the expression of leukocyte adhesion molecules, such as E and P-selectins, the glycoproteins ICAM-1 and VCAM-1, and the chemokine MCP-1, which signals via CCR2 to stimulate migration and infiltration of inflammatory monocytes. LDL deposition promotes the release of M-CSF and GM-CSF, which facilitates the maturation of infiltrating monocytes into macrophages or dendritic cells. LDLs give rise to modified-LDLs (especially oxLDL) that are recognized by macrophage scavenger receptors, such as CD36, LOX-1, and SR-A. These scavenger receptors activate NFκB signaling in macrophages, enhancing the release of proinflammatory cytokines, such as IL1β and TNFα, and leading to the generation of foam cells. Foam cells uptake ox-LDL, and LAL converts cholesterol esters (CE) into free cholesterol (FC) and free fatty acids. FC can be converted into CE by ACAT1 and ACAT2. NCEH1 transforms CE into FC. FC can be transported outside the foam cell by ABCA1, ABCG1, and SR-B1. APCs, such as DCs, process intraplaque oxLDLs and stimulate the adaptive immune response by presenting oxLDL-derived antigens in atheromatous plaques and in secondary lymphoid organs. M1 macrophages, Th1 cells, and some B cell subtypes promote atherosclerosis by the production of proinflammatory cytokines and chemokines, among other mechanisms. In contrast, Bregs, Tregs, M2 macrophages, and tolerogenic DCs suppress inflammation, reducing plaque size and stabilizing atheosclerotic lesions through several mechanisms. Plaque development is also promoted by the differentiation of smooth muscle cells to a proliferating phenotype.

Atherosclerosis is a chronic inflammatory disease of large and medium-sized arteries [20], characterized by endothelial dysfunction and the accumulation of low-density lipoproteins (LDL), immune cells, and necrotic debris in the subendothelial space, resulting in the formation of an atherosclerotic plaque [21,22,23,24]. LDL deposition is more likely in regions with turbulent flow and low shear stress [25] sensed by the vascular endothelium [26]. Turbulent flow modulates endothelial transcriptional and post-transcriptional programs, priming the endothelium for later cytokine activation [27]. Lipid accumulation also stimulates vascular smooth muscle cells (VSMC) and endothelial cells to generate inflammatory mediators and cytokines [28,29], contributing to the initial steps of the atherosclerotic process [30,31]. These changes increase endothelial damage and impair endothelial healing [32]. Endothelial cell activation induces the expression of leukocyte adhesion molecules, such as endothelial-selectin (E-selectin), P-selectin, and the glycoproteins intercellular adhesion molecule-1 (ICAM-1) and vascular cell adhesion molecule-1 (VCAM-1). This is followed by the production of chemokines, such as monocyte chemoattractant protein-1 (MCP-1), which stimulates monocyte migration and infiltration through its receptor C-C chemokine receptor 2 (CCR2). Similarly, interleukin (IL)-8 and fractalkine promote cell migration through C–X–C chemokine receptor type 2 (CXCR2) expressed on leukocytes [27,33,34]. Infiltrating monocytes mature into macrophages in response to macrophage colony-stimulating factor (M-CSF) and granulocyte-macrophage colony-stimulating factor (GM-CSF) [35]. LDLs are modified by oxidation (oxLDL), enzymatic processing, desialylation and aggregation [36]. Macrophages differentiate into foam cells after recognizing and internalizing oxLDLs via an array of scavenger receptors, including scavenger receptor class A (SR-A), CD36, lectin-like oxidized LDL receptor-1 (LOX-1), scavenger receptor for phosphatidylserine and oxidized lipoprotein (SR-PSOX), and scavenger receptor class B type 1 (SR-B1). Moreover, oxLDLs, acting as DAMPs, stimulate TLRs in macrophages, aggravating inflammation in the plaque. Cholesterol efflux from macrophages is regulated by ATP binding cassette transporter A1 (ABCA1) [37]. Therefore, macrophages in the plaque show abnormal lipid metabolism with reduced cholesterol efflux, increased cell death, and reduced efferocytosis, leading to an inflammatory state [11] Figure 2.

OxLDLs also function as immune antigens [38]. DCs and other APCs process intraplaque oxLDLs and stimulate the adaptive immune response by presenting oxLDL-derived antigens in atheromatous plaques and in secondary lymphoid organs [39,40,41,42]. Consequently, CD4^+^ and CD8^+^ T cells are present in the plaque almost as early as monocytes [43] and play essential roles in its development [39,44]. The inflammatory response in the atherosclerotic lesion is also modified by infiltrating B cell subsets [42].

After these initial steps, plaques progress to advanced lesions, composed of lipid droplets, foam cells, macrophages, and lymphocytes [45,46,47]. These cells produce a plethora of cytokines and mediators with important roles in atherosclerotic progression [45,46,47]. Smooth muscle cell growth [48] and the production of collagens, matrix metalloproteinases (MMPs), fibronectin, and elastin also contribute to plaque development [20,49,50]. Among other cell types, macrophages can stimulate production of MMPs that destabilize the plaque by stimulating the production of proinflammatory cytokines [51,52]. Moreover, in advanced plaques, smooth muscle cells, macrophages, and foam cells undergo apoptosis and necrosis, leading to the formation of the necrotic core [53]. Local inflammation in the intima stimulates inflammation in other vessel layers by systemic inflammation in other organs, including adipose tissue and the liver [54,55].

## 4. Innate Immunity

### 4.1. Monocytes

The mononuclear phagocytic system consists of circulating monocytes, monocyte-derived DCs and macrophages, resident macrophages, and DCs [56]. These cells participate in scavenging, inflammation, and anti-pathogen defenses, both in the direct response to foreign agents and in shaping each diverse phase of the inflammatory response [57]. Circulating monocytes, which originate in the bone marrow, are generally found in blood, bone marrow, and the spleen in healthy animals [58]. Circulating monocytes and resident vascular macrophages are the first leukocytes to be recruited to the early atheromatous plaque [59]. Local inflammation is initiated by damaged endothelial cells, which release MCP-1 (also known as C–C motif chemokine ligand 2 (CCL2)). MCP-1 interacts with C–C chemokine receptors (CCR)2 and CCR4 expressed on circulating monocytes, recruiting them to the lesion [60,61,62,63]. Monocyte recruitment also depends on other cytokines and chemokines, including IL-8, CCL3, CCL4, and CCL5 [64,65,66].

Several monocyte subsets are recruited to the atheromatous plaque, with the most common subset in mice being lymphocyte antigen 6 complex high (Ly6C^hi^) monocytes [67]. In humans, the most prevalent monocytes in the plaque are CD14^+^CD16^-^ cells, known as classical monocytes [68,69]. CD14^+^CD16^−^ and Ly6C^hi^ monocytes both express CCR2 [69]. CD14^+^CD16^−^ monocytes have a proinflammatory phenotype [70], and their number is increased in hypercholesterolemic conditions [71]. Human atherosclerotic plaques also contain CD14^+^CD16^hi^ monocytes (nonclassical) and CD14^hi^CD16^+^ monocytes (intermediate). Activated CD14^hi^CD16^+^ monocytes produce large amounts of proinflammatory molecules, such as tumor necrosis factor (TNF) α [72], and have been implicated in atherosclerotic progression [70]. Recruited monocytes are an important systemic source for renewal of tissue macrophages and DCs [73,74], but they are not the only source of plaque monocytes, which can also originate from a local proliferation of monocytes and resident macrophages [75] that maintain their capacity to mature and reach a phagocytic phenotype. Apart from their role as macrophage and DC precursors in atherosclerotic plaques, monocytes can trigger and modulate T-cell responses [76,77], regulate angiogenesis, and exert effector functions as accessory cells in atherogenesis [78]. Increased numbers of circulating proinflammatory monocytes have been found in mouse models of atherosclerosis, such as ApoE^−/−^ mice [79]. Similarly, a high number of intermediate CD14^hi^CD16^+^ circulating monocytes seems to be related to increased atherosclerotic risk in unstable angina patients [80], and upregulation of TLR-4 on intermediate CD14^hi^CD16^+^ monocytes is associated with coronary plaque vulnerability in patients with angina pectoris [80,81].

In mice, circulating monocytes are recruited to the subendothelial space by P and E-selectin-mediated tethering and rolling and ICAM1 and VCAM1-mediated adhesion [34]. Knockdown of VCAM-1 [82] or its inhibition with the antioxidant AGI-1067 [83] results in reduced atherosclerosis in LDLR^−/−^ mice.

Monocyte migration to the subendothelial space depends on signaling via CCR2, CCR5, and CX3C-chemokine receptor 1 (CX3CR1). A blockade of these receptors decreases monocyte recruitment and reduces atheromatous plaque size in ApoE^−/−^ mice [60]. Monocyte recruitment and subsequent atherosclerosis in ApoE^−/−^ mice are also reduced by inhibiting the chemoattractants CCL2, CXCR1, CCR5, and M-CSF, either by pharmacological blockade [60] or genetic inactivation [4,84]. Migrated monocytes differentiate into macrophages that contribute to inflammation and plaque development [79]. In the course of hematopoiesis, the differentiation of monocytes into macrophages is triggered by M-CSF and GM-CSF [85]. Circulating monocytes differentiate in response to stimuli such as inflammation and infection, with inflammation playing an essential role in atherosclerotic development [86].

### 4.2. Macrophages

The early stages of atherosclerosis are characterized by the formation of fatty streaks, and these early lesions contain many macrophages [44]. Macrophages in atherosclerotic plaques maintain local inflammation by producing reactive oxygen species (ROS) and secreting inflammatory cytokines and chemokines, including TNF-α, IL-1β, IL-6, IL-8, and TGF-β, which promote chemotaxis of B and T-cells and macrophages toward the plaque [87]. Intraplaque macrophages have a reduced migratory capacity, acting instead to maintain inflammation and support plaque progression [36,88,89]. Monocytes enter into the arterial intima and differentiate to macrophages [90] but proliferation of artery wall macrophages has been associated with increased plaque development in advanced atherosclerotic lesions [91,92]. Macrophages in lesions internalize and accumulate lipoproteins, converting to foam cells filled with lipid droplets. The accumulation of foam cells drives plaque growth [93]. All phases of atherosclerosis are characterized by macrophage apoptosis and necrosis, which lead to the formation of the necrotic core in progressing plaques [94]. This exacerbates the accumulation of inflammatory cells and impairs the removal of dead cells (efferocytosis), further increasing plaque size and instability [95,96,97].

Macrophages are highly plastic, a property that allows them to hone responses to the specific microenvironment [98,99,100,101]. Moreover, plaque composition and macrophage polarization are interdependent, each influencing the each other [102]. Differentiation of recruited monocytes into macrophages in the plaque is determined by several factors, including the local microenvironment [103], macrophage metabolic state [104,105], gut microbiota-derived metabolites [106], and genetic and epigenetic factors [107].

The classical classification of macrophages envisions two phenotypes, M1 and M2 [108,109,110]. Monocytes give rise to M1 or M2 macrophages in response to exposure to GM-CSF or M-CSF, respectively [111,112,113,114,115], and the predominance of M1 or M2 macrophages in atherosclerotic lesions has been proposed to reflect local M-CSF and GM-CSF concentrations [116]. Complete M1 macrophage differentiation is achieved after exposure to Th1 cytokines, including TNFα and interferon (IFN)-γ, produced upon lipopolysaccharide recognition by TLR or lipoproteins [117].

M1 macrophages are considered proinflammatory cells due to their capacity to secrete abundant proinflammatory factors, such as TNF-α, IL-1α, IL-1β, IL-6, IL-12, IL-18, IL-13, and IL-23, and the chemokines CXCL9, CXCL10, and CXCL11. Proinflammatory macrophages also produce ROS and nitric oxide (NO) [36,118,119,120].

M2 (or alternatively polarized) macrophages can be further divided into four different subsets, M2a, M2b, M2c, and M2d, depending on the activating stimulus [98,99,121,122]. The M2a subset is induced by exposure to the Th2 cytokines IL-4 and IL-13 and expresses high levels of the mannose receptor (CD206) and IL-1 receptor agonist (IL1RN). These cells secrete the anti-inflammatory cytokines IL-10, IL-1, and TGF-β and profibrotic factors, such as fibronectin, that contribute to tissue remodeling [99,121,122]. M2b macrophages are induced by immune complexes, TLR agonists, and IL-1 receptor agonist [99,121,122]. M2c macrophages are induced by glucocorticoids, TGFβ, and IL-10 and produce the anti-inflammatory molecules pentraxin-3 (PTX3), TGFβ, IL-10, and Mer receptor kinase (METK). M2c macrophages are responsible for the removal of apoptotic cells [121,123]. M2d macrophages differentiate in response to TLR signaling via the adenosine A2A receptor; these cells produce large amounts of IL-10 and vascular endothelial growth factor (VEGF) and promote angiogenesis in tumors and atherosclerotic plaques [99,122,124]. M2 macrophages, with the exception of the M2b subset, produce large quantities of anti-inflammatory cytokines, such as IL-10 [125].

Atherosclerotic lesions also contain other macrophage phenotypes. Mox macrophages are inflammatory cells that lack CD163 expression and show reduced phagocytic and chemotactic abilities and an elevated capacity for TLR-2-dependent production of IL-1β and cyclooxygenase-2 (COX-2) in an oxLDL-rich microenvironment [126]. Mox macrophages account for almost a third of the total macrophage content of advanced atherosclerotic plaques in mice [120,127] and play an antioxidant role dependent on their overexpression of Nrf2-related genes, such as *heme oxygenase-1* (*HMOX-1*), *sulforedoxin-1*, and *thioredoxin reductase* [126,127,128]. Mox macrophages were initially described only in mouse models of atherosclerosis [127,129] but have since been identified in humans [130].

Blood vessel injury releases erythrocytes and iron-holding pigments, which can be phagocytosed by macrophages [131,132]. Human atherosclerotic plaques in which neovascularization takes place contain iron deposits that can trigger the differentiation of M(Hb) macrophages [133] (also known as Mhem [134]). M(Hb) macrophages express the scavenger receptor cysteine-rich type-1 protein M130 (CD163) and macrophage mannose receptor 1 (MMR, known as CD206) [135], along with heme-dependent activating transcription factor 1 (ATF1) which induces expression of heme oxygenase 1 and liver X receptor β (LXR-β). The expression of the LXR-β-dependent genes *LXR-α* and *ABCA1* by this macrophage subtype increases cholesterol efflux [133,136], and M(Hb) macrophages have an antiatherogenic role related to their low lipid-loading capacity and anti-inflammatory properties, mediated through the production of IL-10 and apolipoprotein E [133,137,138].

M4 macrophages are produced by stimulation with the chemokine C–X–C motif chemokine 4 (CXCL4) [139,140] and play a proatherogenic role through the production of MMP12 and the promotion of plaque instability [120,141]. M4 macrophages have a lower capacity for phagocytosis than M1 and M2 macrophages [142] and limit the generation of Mhem macrophages [127]. Another intraplaque macrophage subtype is the IL-17A-stimulated macrophage [143].

Macrophages play decisive roles at all stages of atherosclerotic lesion progression [89,144], and intraplaque macrophage subtypes are heterogeneous [145]. Both M1 and M2 macrophages are found in atherosclerotic lesions [120,146,147], with M1 macrophages found in the lesion shoulder, which is the least stable region of the plaque, while both M1 and M2 macrophages are found in the fibrous cap, close to the necrotic core [120,148,149,150]. The production of proinflammatory factors by M1 macrophages results in inflammatory cell recruitment, accelerated plaque development [151], and increased necrotic core formation and plaque vulnerability, leading to thrombotic events [152]. In contrast, M2 macrophages play an anti-inflammatory and atheroprotective role through the inhibition of cell recruitment and tissue remodeling [153]. M2 macrophages also reduce foam cell formation [150] and increase plaque stability [154]. The proinflammatory and anti-inflammatory intraplaque macrophage content can, thus, serve as an index of plaque progression/instability or regression.

LDLs induce proinflammatory macrophage polarization by increasing the production of TNFα and IL-6 and reducing the expression of the anti-inflammatory M2 markers CD206 and CD200R [155]. Modified LDLs promote a stronger proinflammatory phenotype in macrophages upon recognition by TLRs and scavenger receptors like CD36 [156]. OxLDLs also promote a switch in macrophage phenotype from M2 to M1 [157].

Some studies suggest that atherosclerosis’s development might be influenced by macrophage polarization in non-arterial tissues, as described in the epicardial adipose tissue of patients with coronary artery disease [158,159].

### 4.3. Foam Cells

Accumulation of lipoproteins in the arterial intima is a key element in the onset and development of atherosclerosis [160]. Lipoproteins with a diameter below 70 nm include high density lipoproteins (HDL), LDL, intermediate-density lipoproteins (IDL), most very low-density lipoproteins (VLDL), and some chylomicrons, and these biochemical assemblies can cross the endothelium from the blood and enter the arterial intima [161,162], where they are modified by oxidizing agents, proteases, and lipases [163,164,165], generating oxLDLs, acetylatedLDLs, etc. Modification of LDLs also induces their aggregation [165]. These aggregated and modified LDLs can be internalized by VSMCs, DCs, and especially by macrophages, triggering their conversion to foam cells [160,166].

Lipid metabolism in macrophages depends on cholesterol uptake, esterification, and efflux. An imbalance among these processes results in the formation of lipid-dense macrophages, called foam cells [167], and most foam cells are derived from macrophages with a disproportionate influx of modified LDLs and cholesterol esters [168,169]. However, a small fraction of foam cells originate from VSMCs and endothelial cells [170,171]. Monocytes are also important in foam cell formation [172,173]. Macrophages internalize modified or native LDLs after binding by scavenger receptors; eight proteins able to bind modified lipoproteins have been described in macrophages [174,175,176], the best described being SR-A1, CD36, and LOX-1. Scavenger receptors can be modulated by MEKK-2 [177], MAP kinase [177,178], and STA [179]. Macrophages generate cholesterol esters through the action of acyl-coenzyme A: cholesterol acyltransferases (ACATs) [180]. Lipoprotein uptake and cholesterol ester generation are balanced in homeostatic conditions by the hydrolysis of cholesterol esters to free fatty acids and of cholesterol by neutral cholesteryl ester hydrolase 1 (NCEH1) and lysosomal acid lipase (LAL) [181,182]. Cholesterol efflux is mediated by ABCA1, ATP-binding cassette sub-family G member-1 (ABCG1), and scavenger receptor SR-B1 [183]. In disease conditions, this balance is disrupted by the increased macrophage expression of LOX-1 induced by proinflammatory cytokines and by the high levels of oxLDL [181], accompanied by decreased expression of ABCA1 and ABCG1 [184]. These changes result in the intracellular accumulation of cholesterol and the generation of foam cells. Foam cells have less proinflammatory capacity than M1 macrophages in response to the M1-polarizing factors LPS and IFN-γ. In contrast, foam cells develop an anti-inflammatory response similar to M2 macrophages in response to the M2-polarizing signal IL-4 [185]. Indeed, foam cell accumulation in M1-polarizing plaques results in attenuation of macrophage-associated inflammation.

Increased LOX-1 expression in atherosclerosis is also observed in endothelial cells [186], leading to PKC activation. Subsequent activation of RhoA/Rho kinase and protein phosphatase 1 regulatory subunit 14A (PPP1R14A) leads to occludin phosphorylation [187] and cytoskeketal rearrangement [188], respectively. LOX-1 overexpression reduces the expression of the desmosome components desmoglein 1 (DSG1) and desmocollin 2 (DSC2), downregulating desmosomal intercellular contacts [186,189]. Through these mechanisms, atherosclerosis-induced LOX-1 overexpression impairs intercellular interactions, promotes endothelial permeability to oxLDL and its access to the subendothelial space, and enhances foam cell formation [171].

OxLDL and other modified LDLs are internalized by altered phagocytosis [120]. SR-A1 exists in three isoforms. The full-length SR-A1 and shorter SR-A1.1 isoforms are encoded by the *MSR-1* gene and participate in oxLDL recognition and internalization, whereas the lipid-transport-dysfunctional SR-A1.2 isoform acts as an inhibitor of the other two isoforms [190,191]. SR-A1 and SR-A1.1 have high affinity for acetylated LDL and oxLDL [192]. The knockout of *SR-A1* in ApoE^−/−^ and LDLR^−/−^ mice inhibits foam cell formation and reduces atherosclerosis [193,194]. A similar reduction in atherosclerosis is observed in MSR-1^−/−^, ApoE^−/−^ [195], and MSR-1^−/−^ LDLR^−/−^ mice [196,197]. Total or macrophage-specific deletion of SR-As results in fewer atherosclerotic lesions [197]. SR-A1 expression is upregulated by proinflammatory cytokines via NFκB [198] and is downregulated by polyphenols via inhibition of peroxisome proliferator-activated receptor γ (PPARγ) and by curcumin via calpain-associated ubiquitination and degradation [199].

CD36 is a member of the B family of scavenger receptors [200,201]. OxLDL-dependent CD36 production regulates inflammation through the induction of the TLR4/TLR6 complex assembly, NFκB activation, and chemokine release [202]. Accordingly, CD36 deficiency in macrophages diminishes cytokine production [203]. CD36 inhibition reduces oxLDL content in the arterial wall [204,205,206,207].

In macrophages, CD36 levels can be upregulated by curcumin-induced expression of nuclear factor (erythroid-derived 2)-like 2 (NFE2L2) [208], whereas in monocytes, CD36 is increased by LPS via upregulation of AP-1 transcription factors [209] and by palmitate [210]. CD36 can be inhibited by ceramides in monocytes [211] and by plant antioxidant in macrophages [212,213,214]. *CD36* knockout in ApoE^−/−^ mice protects against atherosclerotic lesion development only in males [215]. In contrast, CD36^−/−^-ApoE^−/−^ females show a relative increase in the number of atherosclerotic lesions [216].

LOX-1 is highly expressed in atherosclerotic plaques in humans [217] and in macrophages during atherosclerosis [181], accounting for 40% of oxLDL internalization [218]. LOX-1 is induced in macrophages by proinflammatory cytokines [181], oxLDL [219], LPS [220], advanced glycation end-products [209], and ROS [221]. In mouse models of atherosclerosis, *LOX-1* knockout decreases disease and inflammation, whereas LOX-1 overexpression has the opposite effect [220,221,222,223].

The accumulation of cholesterol esters in macrophages leads to the generation of foam cells. The transformation of cholesterol to cholesterol esters is catalyzed by acetyl-CoA acetyltransferase (ACAT1) [180], and the reverse process is mediated by NCEH [181,182].

Atherosclerosis in LDLR^−/−^ mice is aggravated by macrophage-restricted depletion of ACAT1 [224]. In contrast, systemic ACAT1 deficiency ApoE^−/−^ and LDL^−/−^ mice does not affect atherosclerosis, but these mice develop dermal xanthomas and form cholesterol deposits in the brain [225]. Pharmacological inhibition of ACAT1 increases plaque formation in mouse and rabbit models of atherosclerosis [226]. ACAT1 activity or expression can be modulated by several molecules, such as the non-specific inhibitor F-1394 [227], the intestinal hormone Ghrelin via PPARγ [228], protein kinase A (PKA) via incretin hormones [229], dipeptidylpeptidase 4 (DDP4) via the incretin hormone glucagon-like peptide-1 (GLP-1) [230], insulin via CCAAT/enhancer-binding protein α (C/EBPα) and extracellular signal-regulated kinase (Erk), p38MAP kinase, Jnk [231], and leptin via janus-activated kinase 2 (Jak2)/phosphatidylinositide 3-kinase (PI3K) [232].

NCEH hydrolyzes cholesterol esters to release free cholesterol [182] that can be exported from the cell. In mouse models, NCEH inhibition increases atherosclerosis [233], whereas NCEH overexpression diminishes the lesion’s necrotic core [234]. NCEH overproduction in macrophages also reduces cholesterol esters [235]. The NCEH1 isoform accelerates atherosclerosis in ApoE^−/−^ mice [236]. NCEH and NCEH1 inhibit foam cell generation [237,238].

Cholesterol efflux is mediated by the transporters ABCA1 and ABCG2 and the scavenger receptor SR-B1, and by passive membrane diffusion with simultaneous genetic disruption of ABCA1; and SR-B1 potentiates foam cell formation but has no effect on lesion development [239]. In LDLR^−/−^ mice, ABCA1 overexpression in the liver leads to the accumulation of proatherogenic LDL and enlarged aortic atherosclerotic lesions [240].

The effect of ABCG1 deletion is antiatherogenic [241] or moderately proatherogenic [242], depending on the study. SR-B1 overexpression has an atheroprotective effect, whereas its deletion produces a proatherogenic phenotype, demonstrating the antiatherogenic role of SR-B1 [243].

ABCA1 is regulated by transcription factor liver X receptor α (LXRα) [244], the flavonoid quercetin via the PPARγ/LXRα pathway [245], proteasome inhibition [246], and ApoA-1 [247]. Foam cell formation is reduced by ABCA1 upregulation by C–X–C motif chemokine 5 (CXCL5) [248], as well as cAMP, sterols, and PPARγ agonists [249]. ABCA1 expression is negatively regulated by unsaturated free fatty acids via PKCδ-dependent phosphorylation [250,251] and by IL-12 and IL-18 via activation of the zinc finger protein ZNF202 [252]. Macrophage ABCA1 and ABCG1 can be upregulated by the monoterpenoid cineol [253] and by olive-oil [254]. ABCA1 and ABCG1 levels are increased by the gut microbiota metabolite—protocatechuic acid (PCA), acting via miR-10b [255]. ABCG1 and SR-B1 levels are increased by caffeic and ferulic acids [256]. LXRα and SR-BI are enhanced by resveratrol and 13-hyroxy linoleic acid via PPARγ [257,258]. ABCA1, ABCG1, and SR-B1 are reduced via LXRα by the metalloproteinase pappalysin-1 (PAPPA), which hydrolyzes insulin-like growth factor-binding proteins (IGFBP) [259].

The accumulation of cholesterol crystals provokes the production of pro-inflammatory cytokines by M1 macrophages via the caspase-1-activating NLRP3 inflammasome [260]. Moreover, cholesterol esters induce the M1 phenotype by activating TLR4 and inducing the NF-κβ pathway [261]. A major product of cholesterol ester oxidation, 9-oxononanoyl-cholesterol, increases the release of TGF-β and promotes the generation of anti-inflammatory macrophages [262]. In addition, conjugated linoleic acid promotes M2 polarization by augmenting IL-10 production [44,263]

### 4.4. Dendritic Cells

DCs link the innate and adaptive immune systems by presenting antigens to T cells. Immature DCs patrol tissues under physiological conditions. Upon activation, maturing DCs increase the expression of MHC class II (MHCII) and the costimulatory molecules CD80, CD86, and CD83 [264,265] and migrate to draining lymph nodes, where they stimulate the adaptive immune response by priming T cells and secreting cytokines [266]. Key features of DCs include stellate morphology, high expression of MHCII, and the capacity to take up, process, and present antigens, such as those derived from apolipoproteins [267].

Several DC subsets derive from a specific precursor population of common dendritic progenitors (CDP). CDPs give rise to plasmacytoid DCs (pDCs) in bone marrow or generate pre-DCs that circulate in blood and give rise to classical DCs (cDCs) in lymphoid and non-lymphoid organs. cDCs can be classified as cDC1 and cDC2 [265,268,269,270,271]. pDCs and cDCs are induced by the growth factor fms-like tyrosine kinase 3 ligand (Flt3L) [41]. DCs can also originate from circulating monocytes after their migration to tissues, where they differentiate to macrophages or monocyte-derived DCs through the action of M-CSF or GM-CSF, respectively; these monocyte-derived DCs are considered part of the mononuclear phagocyte system (MPS) [272,273]. Monocyte-derived DCs express CD11c and MHCII, and these markers have been widely used in studies of the role of DCs in atherosclerosis; however, some monocytes and macrophages also express these markers [274]. Following the nomenclature used by other authors [41], herein we refer to CD11c^+^MHCII^+^ DCs as APCs, and use pDC and cDC for more specific DC populations.

During atherosclerosis, endothelial cells enter a state of chronic activation [275], leading to the recruitment of monocytes into the subendothelial intima. Conditions in the intraplaque microenvironment trigger the generation of monocyte-derived DCs. The role of these DCs in atherosclerosis is unclear. A proatherogenic role in the early phases of atherosclerosis has been proposed, [276] as has as an atheroprotective function [88].

CD11c^+^MHCII^+^F4/80^−^ APCs have been detected in atherosclerosis-prone areas of the mouse aorta before lesions develop [277,278]. CD11c^+^ APCs migrate from atherosclerotic plaque upon blockade of CCL19 and CCL21, in a process dependent on CCL7 [279,280]. CD11c^+^MCHII^+^ DCs have been found in atheromatous plaques in mice [281,282,283] containing Flt3-dependent CD103^+^ and CD11b^+^ CD172a^+^ cDCs [281,282] and monocyte-derived CD11b^+^ DCs, both of which are M-CSF-dependent [281] and CD64-expressing [282]. In the early stages of plaque formation, CD11c^+^ APCs can take up lipids and contribute to foam cell formation [276]. DCs in advanced atherosclerotic lesions show the activation of markers, including the costimulatory molecules CD83 and CD86 and cytokines [74,284].

Antigen presentation by DCs has been described in secondary lymphoid organs and in the atheromatous plaque [41,285]. Isolated aortic CD11c^+^MHCII^+^ APCs induce T cell proliferation in vitro [278,286] and stimulate T cell production of TNF-α and IFN-γ [287]. The importance of antigen presentation by APCs in atherosclerosis has been revealed by several studies. In *LDLR*^−/−^ mice, abrogation of the invariant chain of CD74, a protein involved in MHCII-peptide complex formation [288], reduces T cell activation and atherosclerosis; however, in *ApoE*^−/−^ mice, the inability to present antigens on MHCII increases atherosclerosis by reducing the pool of atheroprotective Tregs and increasing proatherogenic CD8^+^ T cells [289]. The reason for this discrepancy is unknown, but an atheroprotective role of antigen presentation via MHCII is supported by the finding that TLR-activated CD11c^+^ DCs promote Treg development and function in association with increased atherosclerosis [290]. Additionally, a cDC-specific loss of MHCII causes colitis and reduced Treg activation [291,292]. Atherosclerosis is also affected by the modulation of other key molecules in DCs’ function. Lack of TGFβ type II receptor signaling in CD11c^+^ APCs promotes atherosclerosis in *ApoE*^−/−^ mice [293]. Loss of (HIF)-1α in CD11c^+^ APCs in *LDLR*^−/−^ mice increases proatherogenic T cell infiltration and the expansion of atherogenic Th1 cells [294]. The ablation of MyD88, a TLR adaptor, in CD11c^+^ APCs produces a reduction in Tregs [290]. Abrogation of the mechanosensitive Kruppel-like factor 2 (KLF2) in CD11c^+^ APCs promotes surface localization of the costimulatory molecules CD40 and CD86 and increased T cell proliferation and apoptosis. After *Klf2*^−/−^ bone marrow transplant to *LDLR*^−/−^ mice, the absence of KLF2 increases the number of DCs in lesions, enhances T cell activation and cytokine production, and increases atherosclerotic lesions’ size [295]. APCs also mediate tolerogenic responses by reducing effector T cell functions and enhancing Treg functions [296,297]. In *LDLR*^−/−^ mice, oxLDL-loaded, bone–marrow-derived DCs reduce atherosclerotic lesion size by inhibiting the Th1 response [298], while ApoB100-loaded, bone–marrow-derived DCs achieve the same effect by increasing Treg responses [299].

#### 4.4.1. cDCs

Investigation of the role of cDCs in atherosclerosis has yielded conflicting results. Atherosclerotic plaque size is unaffected in *LDLR*^−/−^ mice, reconstituted with bone marrow depleted of cDCs by the insertion of diphtheria toxin receptor (DTR) into the cDC-specific Zbtb46 locus [300]. However, CD103^+^ DC depletion in *LDLR^−/−^Flt3^−/−^* mice shows the depletion of aortic Tregs and increased atherosclerosis [301]. Plaque size is unaffected in LDLR^−/−^ mice reconstituted with Baft3^−/−^ bone marrow [302] and in *LDLR^−/−^Batf3^−/−^* mice [303]. However, experiments in *ApoE^−/−^ Batf3^−/−^* mice support a proatherogenic role and elevated Th1 stimulation capacity for Batf3-dependent DCs [304]. These disparities likely reflect the different mouse models used. A proatherogenic function has been described for DNGR1^+^ CD8α^+^/CD103^+^ DCs [305].

#### 4.4.2. pDCs

Depletion of pDCs in atherosclerotic mouse models has been achieved using a number of antibodies against bone marrow stromal cell antigen 2 (BST2), resulting in enhanced atherosclerosis in *LDLR*^−/−^ mice [306] and reduced atherosclerosis in ApoE^−/−^ mice [307,308]. Injection of diphtheria toxin (DT) to deplete pDCs in LDLR^−/−^ mice, reconstituted with γ-irradiated BDCA2-DTR bone marrow, results in increased atherosclerosis [309]. In contrast, atherosclerosis is unaffected in DT-treated *ApoE*^−/−^ BDCA2-DTR mice [310].

An atheroprotective effect was found upon CD11c-specific deletion of the transcription factor E2-2/Tcf4 in pDCs or impairment of MHCII antigen presentation in pDCs [311], indicating a proatherogenic role of pDCs in MHCII-dependent antigen presentation via T cell responses in atherosclerosis.

Human studies suggest a correlation between low circulating levels of cDCs and pDCs and peripheral artery disease [312], and DC accumulation [313] and activation [314] is associated with plaque vulnerability.

#### 4.4.3. Regulatory DCs

Regulatory DCs can present antigens but express low levels of costimulatory molecules and proinflammatory cytokines, while expressing higher levels of anti-inflammatory cytokines; moreover, regulatory DCs are refractory to maturation signals [315,316]. Regulatory DCs promote immune tolerance by reducing the expression of costimulatory molecules, increasing the expression of inhibitory molecules, such as indoleamine 2,3-dioxygenase (IDO), inhibiting the production of the proinflammatory cytokines IL-12 and TNFα, and promoting the induction of the anti-inflammatory cytokines IL10 and TGFβ [317]. Regulatory DCs mediate depletion and anergy of proinflammatory effector T cells [318,319] and the generation and expansion of atheroprotective Tregs [320].

Self-antigen recognition plays an important role in the chronic inflammation associated with atherosclerosis [321]. The accumulation of native LDLs, oxLDLs, and apolipoprotein B100 (ApoB100) in the vessel wall attracts immune cells and triggers chronic inflammation. The loading of DCs with oxLDL and ApoB100 diminishes the generation of proinflammatory cytokines and promotes the production of Tregs [298,299].

DC tolerance is generated by anti-inflammatory molecules, such as IL-10 and TGFβ, and immunosuppressive enzymes, such as IDO [322]. Adoptive transfer of oxLDL-loaded DCs exacerbates atherosclerosis [309,323], while intravenous transfer of tolerogenic ApoB100-loaded and IL10-treated DCs attenuates atherosclerosis in hypercholesterolemic LDLR^−/−^ mice expressing human ApoB100 [299].

Apoptotic cells are removed through efferocytosis, and the impairment of this process in atherosclerotic plaques increases inflammation. Intravenous administration of oxLDL-induced apoptotic DCs to LDLR^−/−^ mice reduces atherosclerosis and increases plaque stability by increasing CD103^+^ tolerogenic DC and Treg numbers and reducing the numbers of Ly6C^hi^ monocytes and the level of circulating CCL12 [324]. Further studies are needed to determine the role of tolerogenic DCs in atherosclerosis’s onset and development.

## 5. Adaptive Immunity

Adaptive immunity is a highly precise and lifelong immune response that plays essential roles in distinguishing foreign from self-antigens. Adaptive immunity is mainly mediated by T and B cells, which precisely recognize antigens through the specific receptors expressed on their surfaces, the T-cell receptor (TCR) and B-cell receptor (BCR) [325].

T cells are classified according to membrane and intracellular markers. They express the αβ or γδ TCR, CD3, and one of the coreceptors CD4 or CD8. The TCR-CD3 complex recognizes antigens presented in the context of major histocompatibility complex molecules (MHC or human leukocyte antigen (HLA) in humans) by an APC [325].

B cells produce antibodies, act as APCs, and release cytokines. B cells are categorized according to the expression of the cell-lineage marker CD19 and a variety of surface and intracellular proteins; the distinct BCRs they express; and their production of antibodies and cytokines [325].

APCs are able to present antigens to cognate naïve CD4^+^ and CD8^+^ T cells [326]. These antigens include non-self-antigens and self-antigens, including ox-LDLs and heat shock protein 60 (HSP 60) [327]. Upon activation, CD4^+^ T cells proliferate and differentiate into specialized effector T helper (Th) cells, whereas activated CD8^+^ T cells proliferate and differentiate into CD8^+^ cytotoxic T lymphocytes (CTL) [328]. Naïve CD4^+^ T cells can differentiate into various cell subsets, including effector T cells (T helper 1 (Th1), Th2, and Th17) and regulatory T cells (Treg) [329]. T-cell differentiation into varied Th subsets depends on the type of antigen encountered, the TCR signal intensity, and the local cytokine milieu [330,331,332]. These factors mediate Th polarization in atherosclerotic lesions [333].

### 5.1. CD4^+^ T Cell

Th phenotypes are classified by the differential expression of surface molecules, transcription factors, and effector cytokines [332]. Th1 cells are characterized by the release of large amounts of IFN-γ and IL-2 and the expression of the master transcription factor T-bet. Th2 cells produce IL-4, IL-5, and IL-13 and express the master transcription factor GATA-3. Th17 produce the cytokine IL-17 and express the transcription factor RORγt [334]. Tregs are defined by the expression of the transcription factor forkhead box 3 (Foxp3) and the extracellular marker CD25.

Although macrophages account for most inflammatory cells in atherosclerotic lesions, T and B cells play an essential role in atherosclerotic plaque development through by their capacity to control immune responses during disease onset and progression [335,336]. Atherosclerotic plaques have been found to contain CD4^+^ and CD8^+^ T cells, B cells, NKT cells, and follicular helper T cells [2,87,337,338,339]. T cells account for 10% of all cells in human plaques, with 70% of them being CD4^+^ T cells and most of the remaining 30% being CD8^+^ T cells [46]. The most abundant CD4^+^ T cells in the plaque are Th1 cells, but Th2, Treg, and Th17 cells have also be found, as have TCRγδ^+^ T cells and NKT cells [340,341,342,343].

Global CD4^+^ T cell abrogation in ApoE^−/−^ mice confers atheroprotection [344,345], whereas plaque size is increased by adoptive transfer of CD4^+^ T cells from ApoE^−/−^ mice or modified-LDL-reactive CD4^+^ T cells [8]. However, the total abrogation of CD4^+^ T cells results in the absence of proinflammatory and anti-inflammatory cell populations with opposite influences on atherosclerosis.

#### 5.1.1. Th1

Th1 cells play a proatherogenic role in atherosclerosis [45,327,333,346,347,348]. OxLDLs increase antigen presentation by DCs through HSP60, enhancing T-bet expression, Th1 polarization, and IFN-γ production [349]. Moreover, activated macrophages in atherosclerotic lesions produce IL-12 and IL-18, which also induce Th1 polarization and IFN-γ production [349]. The IL-12–IL-18–T-bet–IFN-γ pathway is a powerful proinflammatory stimulus that promotes and accelerates lesion development, and atherosclerosis is reduced by disruption of the IL12 gene in ApoE^−/−^ mice [350] or by functional blockade of IL-12 with anti-IL-12 antibodies [351], accompanied by increased plaque stability, as indicated by augmented collagen levels. Atherosclerosis in ApoE^−/−^ mice is also reduced and plaques are stabilized by IL-18 blockade with an IL-18 binding protein [352] or by genetic deletion [349].

The Th1 lineage-specific transcription factor T-bet, a member of the of the T box family, promotes Th1 differentiation [353] by binding to the IFN-γ promoter, stimulating IFN-γ production [354]. T-bet deficiency slows atherosclerosis development and increases production of the atheroprotective Th2 cytokines IL-4, IL-5, and IL-10, and IgM antibodies [346]. T-bet also promotes atheroprotection in cooperation with Foxp3 by increasing Treg activity [355].

Th1 cells produce high levels of IFN-γ [333]. IFN-γ promotes plaque development, as shown in ApoE^−/−^ mice by the proatherogenic effect of recombinant IFN-γ injection [356] and the atheroprotective effect of *Ifng* gene knockout [357]. IFN-γ stimulates the IFN-γ cell surface receptor complex (IFN-γR), activating janus kinases (JAK) and recruiting and activating STAT1 (signal transducer and activator of transcription 1), which translocates to the nucleus and stimulates the transcription of IFN-γ target genes, such as *MCP-1* and *ICAM-1* [358]. Inhibition of STAT1’s activity in LDLR^−/−^ mice is atheroprotective, confirming the importance of the IFN-γ–JAK–STAT1 pathway during lesion progression [179,359]. IFN-γ modulates the recruitment of immune cells by inducing endothelial cell expression of ICAM-1 and VCAM-1 [360,361] and by promoting foam cell formation through the modulation of key genes involved in cholesterol metabolism, including ABCA1 and ACAT1 [362], and SR-A and SR-SPOX [363,364,365]. IFN-γ also stimulates CCR5 production in atherosclerosis [343,366].

The proatherogenic effect of Th1 cells in atherosclerosis appears to involve an important role for the chemokine receptor CCR5, a well-known HIV-1 co-receptor, through the T cell recruiting action and other effects of its ligands: macrophage inflammatory protein 1α (MIP-1α, CCL3), MIP-1β (CCL4), and RANTES (regulated on activation normal T cell expressed and secreted, CCL5) [348]. The naturally-occurring human variant CCR5delta32 downregulates *CCR5* gene function. The CCR5delta32 allele has been linked to protection against coronary artery disease [367,368] and heart disease [369] and a lower risk of myocardial infarction [370,371]. However, this protective effect has not been observed in other studies in diverse populations [372,373,374,375,376]. Mouse studies show that CCL5 receptor antagonism [90,377,378,379] and genetic depletion [65,366,380,381,382,383] exacerbate atherosclerosis. The CCR5 blocking antibody maraviroc has been used in HIV patients to block viral entry to CD4 T cells. CCR5 blockade with this antibody also reduces atherosclerosis in mouse models and HIV patients [384,385]. Deletion of CCL5 in ApoE^−/−^ mice reduces atherosclerosis in a CCR5-dependent-manner [386], and in [44AANA47]-RANTES, which prevents CCL5 ligation with glycosaminoglycans, reduces atherosclerosis, and increases plaque stability in LDLR^−/−^ mice [387]. In addition, disruption of TGF-β signaling in CD4^+^ T cells of ApoE^−/−^ mice promotes atherosclerosis by enhancing the Th1 response [388].

#### 5.1.2. Th2

Th2 differentiation is triggered by IL-4 via STAT6-induced GATA-3 expression [389]. Th2 cells are characterized by the production of IL-4, IL-5, and IL-13 and the promotion of B cell-mediated responses. The role of Th2 cells in atherosclerosis remains controversial [327,333]. An atheroprotective role is supported by the association of elevated numbers of circulating Th2 cells with less severe atherosclerosis and a lower risk of acute myocardial infarction in women [390]. Moreover, Th2 cells counteract the proatherogenic effects of IFN-γ-producing Th1. The Th2 cytokines IL-5 and IL-3 appear to be atheroprotective [391,392,393] through their capacity to augment collagen deposition, diminish monocyte recruitment, and potentiate M2 polarization [393,394]. ApoB vaccination promotes Th2 responses that reduce atherosclerosis [395]. However, experimental manipulation of the signature Th2 cytokine IL-4, which inhibits Th1 responses [350,396], has produced conflicting results. Adoptive transfer of IL-4^−/−^ bone marrow to LDLR^−/−^ mice significantly reduces atherosclerosis [396], but similar studies in IL4^−/−^ plus *ApoE^−/−^* or *LDLR^−/−^* double knockouts showed no effect on atherosclerotic plaque development, and IL-4 administration provided no protection against atherosclerosis [397]. Moreover, IL-4 increases a macrophage’s expression of the proatherogenic molecules CD36 [398] and SR-A [399], and of VCAM-1 [400,401], MMP1 [402], and MCP1 [403].

Recent studies suggest that indirect modulation of the Th2 response is atheroprotective. *MHCII^−/−^ApoE^−/−^* double knockout mice show aggravated atherosclerosis and reduced levels of Th2 cytokines in plasma [289]. Similarly, CCL1^−/−^ApoE^−/−^ mice show increased atherosclerosis and an elevated splenocytic Th1:Th2 ratio [404], whereas IL-12p35^−/−^ApoE^−/−^ mice show reduced atherosclerosis and a reduced Th1:Th2 ratio [405]. Notably, these three mouse models all show a significant atheroprotective role of Tregs. In contrast, the Th2 response appears to aggravate atherosclerosis in an asthmatic ApoE^−/−^ mouse model [406].

#### 5.1.3. Th17

Th17 cells have a role in the protection against extracellular pathogens [407]. Th17 differentiation is induced by IL-6 and TGF-β, which mediate the activation of STAT3 and the production of the Th17 signature transcription factor retinoic acid-related orphan receptor γT (ROR γT). Th17 cells and their signature cytokines have been linked to autoimmune diseases [408] and to atherosclerosis [409,410,411,412,413].

Th17 cells are found in human atherosclerotic plaques [414] but appear to have proatherogenic and antiatherogenic properties [409,410], possibly depending on animal model or methodological approach used and reflecting specific responses of Th17 cells to environmental cues [413]. The pathogenic action of Th17 cells in atherosclerosis depends on their capacity to produce proinflammatory factors, such as IL-6, IFN-γ, and GM-CSF [415,416], and some pathogenic Th17 cells are derived from Tregs that lose FOXP3 expression and immunosuppressive properties [417].

Th17 polarization is triggered by several cytokines [410]. IL-23 enhances pathogenic and proinflammatory effects of Th17 cells in humans [418,419,420], and IL-23 blockade impedes Th17 cell production of IFN-γ and GM-CSF [421]. Pathogenic Th17 cells are also potentiated by IL-1β and TGF-β3 [422] and are differentiated into atheroprotective IL-10-producing cells by TGF-β1 in the intestine [423].

Th17 cytokines play an important role in atherosclerosis. Th17 cells secrete IL-17A, IL-17F, IL-22, and IL-23 [424,425,426,427]. IL17 stimulates the NF-κB, ERK1/2, CCAAT/enhancer-binding protein β (C/EBPβ), and C/EBPδ signaling pathways [428]. This induces the production of proinflammatory cytokines, including TNFα, IL-1β [429,430], IFN-γ [431], IL-6, IL-8 [432], and GM-CSF [433] in various target cells, such as endothelial cells, smooth muscle cells, macrophages, and Th1 cells [428].

IL-17A can be produced not only by Th17 cells but also by CD8^+^ T cells, γδ T cells, invariant natural killer T cells (iNKT), NKT cells, natural Th17 cells, lymphoid tissue inducer (LTi) cells, group 3 innate lymphoid (ILC3) cells, macrophages, neutrophils, and mast cells [432]. The role of IL-17A in atherosclerosis appears to be complex, with both proatherogenic and antiatherogenic roles reported [14,414,429,434,435]. A proatherogenic role is supported by the capacity of IL-17A to induce VSMC production of proinflammatory factors, such as IL-6, CXCL8, and CXCL10 [436], whereas an anti-inflammatory role is suggested by its ability to inhibit the action of VCAM-1 and adhesion molecules on fibroblasts and VSMCs [437] and by its capacity decrease the production of proatherogenic IFN-γ [438].

Several studies have reported that genetic abrogation of *IL-17A* or its receptor in atherogenic mouse models reduces atherosclerotic lesions and plaque vulnerability, supporting a proatherogenic role for IL-17 [429,439,440,441,442]. Another study showed that antibody blockade of IL-17A in ApoE^−/−^ mice reduces plaque vulnerability [429]. However, another study indicated that IL17A is atheroprotective and favors plaque stability in LDLR^−/−^ mice [435]. VSMC-dependent release of IL-17C plays a proatherogenic role by promoting the recruitment of proinflammatory IL-17A-expressing Th17 cells to atherosclerotic plaques [443].

IL-22 is a member of the IL-10 family produced by Th17, Th22, cells, γδ T cells, NKT cells, and ILCs [444,445,446] that has been linked to proinflammatory and anti-inflammatory roles [412,447,448,449,450]. IL-22 has been detected in atherosclerotic lesions [451] and reduces cholesterol efflux from macrophages by reducing the expression of the cholesterol efflux transporter ABCG1 [452] and promotes the production of MMP-9 and the proinflammatory cytokines IL-1β, IL-6, and TNFα [412]. IL-22^−/−^ApoE^−/−^ mice show reduced plaque size and increased VSMC integrity [453]. Intriguingly, IL-23 and IL-22 reduce atherosclerosis by repressing proatherogenic microbiota [454].

#### 5.1.4. Tregs

Tregs suppress a wide range of immune cells, including CD4^+^ and CD8^+^ T cells, B cells, and NKT cells and drive DCs and macrophages toward a more tolerogenic phenotype.

Most Tregs express the master regulator of Treg development and function FOXP3, which is used as a marker for their identification. Natural Tregs (nTreg), which are generated in the thymus, mainly mediate tolerance to self-antigens through the expression of IL-10 and TGF-β [333]. nTregs are positive for the markers FOXP3^+^CD4^+^CD25^+^ [455,456]. Inducible Tregs (iTregs) are prominently implicated in pathogen tolerance and are generated from naïve T cells in the periphery [457]. iTregs may or may not express FOXP3 and present a variety of phenotypes, including Foxp3^+^Tregs; IL-10-producing T regulatory type 1 (Tr1) cells, which lack FOXP3 and CD25 expression [458]; and TGF-β-producing T helper type 3 (Th3) cells. Th3 cells intervene in oral tolerance [459], express CD25 and Foxp3, and mediate suppression of Th1 and Th2 proliferation primarily by the release of TGF-β, but not IL-10 [456,460].

Treg cell generation and function is promoted by CD11c^+^CD103^+^ DCs [290] and signaling from IL-10 and TGF-β [461]. Tregs play an important role in the suppression of immune responses, self-tolerance, and homeostasis [462] by regulating the immune balance.

Tregs exert their immunoregulatory and suppressive activity through several mechanisms [463,464,465]. One prominent mechanism is contact-dependent cell-mediated inhibition, and Tregs express suppressive surface molecules, such as T-lymphocyte–associated antigen-4 (CTLA-4) and programmed cell death-1 (PD-1). CTLA-4 downregulates APC function and T cell activation by diminishing CD80 and CD86 expression in the APC and by blocking the co-stimulatory interaction between CD80/CD86 in the APC and CD28 in the T cell [466]. Tregs can mediate effector cell eradication by the production of granzyme B [467] or by the generation of the tumor necrosis factor-related apoptosis inducing ligand (TRAIL)/death receptor 5 (DR5) and galectin-1, which promote effector T cell apoptosis [468,469]. Tregs also secrete the immunosuppressive cytokines TGF-β, IL-10, and IL-35 [463].

FOXP3 expression and Treg generation and function are induced by TGF-β [470]. TGF-β reduces plaque size and increases plaque stability by inhibiting the recruitment and activation of proinflammatory cells and by increasing VSMC numbers and promoting collagen accumulation [388]. In ApoE^−/−^ mice, disruption of TGF-β signaling enhances atherosclerosis, whereas TGF-β overexpression reduces atherosclerotic lesion vulnerability and atherosclerosis [388].

IL-10 plays a protective role in atherosclerosis by inhibiting Th1 differentiation and decreasing T cell and macrophage accumulation and by diminishing the production of proinflammatory cytokines [471,472]. In ApoE^−/−^ mice, Tr1 cells, among other cell types, reduce inflammation and plaque lesions by producing IL-10 and reducing the generation of IFN-γ [473].

IL-35, a member of the IL-12 cytokine family, binds to IL-35R and stimulates STAT1 and STAT4 in T cells and endothelial cells [474,475,476,477] and STAT1 [478] in B cells [475,479,480,481,482]. IL-35 suppresses Th1 and Th17 cell proliferation and function while promoting Treg and regulatory B cell activity [477,482]. IL-35 is found in atherosclerotic lesions [483,484], and its circulating level is reduced in stable angina pectoris patients [484]. Treatment with IL35 reduces atherosclerosis in ApoE^−/−^ mice [485].

Nevertheless, the role of IL-35 is complicated by its sharing of subunits with other IL-12 family members, including subunit IL-12A with IL-12 and subunit EBI3 with IL-27. EBI3 deficiency in LDLR^−/−^ mice reduces atherosclerosis [486], although other studies indicate a proatherogenic role for IL-27 [486,487]. Therefore, further studies are needed to define the role of IL-35 and IL-27 in atherosclerosis.

Tregs mediate immunosuppression by disruption metabolism. Tregs deplete IL-2 from the media [488] and express CD39 and CD73, which then hydrolyze extracellular ATP to produce pericellular adenosine [489,490]. This adenosine activates the adenosine A2A receptor, which potentiates Tregs and inhibits effector T cell functions [491].

Tregs are found in atherosclerotic lesions [492]. ApoE^−/−^ mice fed a hypercholesterolemic diet have fewer Tregs and more atherosclerosis than counterparts fed a normal diet [493]. Atherosclerosis has been linked to a variety of Treg phenotypes [463,494]. Several studies demonstrate a protective role for Treg subsets [463,494,495]. For example, disruption of CD4^+^CD25^+^ Tregs with anti CD25 antibodies in ApoE^−/−^ mice accelerates the development of atherosclerosis lesions and increases plaque vulnerability [496]. Atherosclerosis is also increased in LDLR^−/−^ mice reconstituted with bone marrow depleted of FOXP3^+^ Tregs using a DT-based procedure [497,498]. Conversely, atherosclerosis is prevented by inducing Tregs with anti CD3 [499,500] and IL-2/anti-IL2 [501] or both monoclonal antibodies [502]. Protection against atherosclerosis is also achieved by reducing the effector T cell to Treg ratio with anti-CD3 monoclonal antibodies [503] or by exposure to ultraviolet B [504].

We recently reported that atherosclerosis is increased in ApoE^−/−^ mice upon inhibition of Treg recruitment in early atherosclerotic plaques by the genetic disruption of *CCL1*, while a similar effect is observed in LDLR^−/−^ mice upon monoclonal antibody blockade of the CCL1-receptor CCR8, suggesting the importance of Treg recruitment in atherosclerosis development [404].

Impairment of Treg function may contribute to the acceleration of atherosclerosis. Tregs from ApoE^−/−^ mice have lower inhibitory Treg function than C57BL/6 mice, similar to the impaired Treg function observed in acute coronary syndrome patients [505]. Similarly, CCL-1 genetic disruption reduces Treg inhibitory capacity in ApoE^−/−^ mice fed a high-fat diet [404].

The importance of Treg abundance and the effector T cell to Treg ratio in atherosclerosis is also supported by human studies. This is suggested by to the impaired Treg function observed in acute coronary syndrome patients [505] and the low abundance of peripheral Treg in patients with acute coronary syndrome or stable angina [506,507,508,509] and the higher effector T cell to Treg ratio observed in patients with coronary artery disease than in healthy individuals [510]. Treg numbers in patients with non-ST segment elevation acute coronary syndrome are reduced by apoptosis promoted by oxLDLs [506,509].

These data highlight the importance of the abundance and function of several Treg subsets and Treg-related cytokines in the development of atherosclerosis.

### 5.2. CD8 T Cells

CD8 T cells play a prominent role in the defense against intracellular pathogens. These T cells recognize antigen via the TCR in the context of ubiquitously expressed MHC class I molecules (MHC-I; HLA-A, B, or C in humans). Upon antigen recognition, naïve CD8^+^ T cells are activated, proliferate, and differentiate into CD8^+^ CTLs [511]. CD8 T cells play effector functions through the release of proinflammatory cytokines [511], such as TNF-α, which can induce apoptosis, and IFN-γ, which promotes MHC-I upregulation and further promotes the inflammatory response. CD8 T cells promote Fas receptor mediated-apoptosis of the target cell through the expression of Fas ligand and target cell lysis through the release of granzymes and perforin [512].

Some studies show no effect or a protective effect of CD8 T cells in atherosclerosis, whereas others report a proatherogenic effect [513]. A neutral or protective action is supported by unaltered atherosclerosis in ApoE^−/−^ mice lacking antigen peptide transporter 1-(TAP1)-dependent MHC-I antigen presentation, despite the low number of CD8^+^ T cells in these animals [514]. Moreover, atherosclerosis in ApoE^−/−^ mice is also unaffected by genetic disruption of CD8a [344]. However, increased plaque development was reported in a MHC-I deficient mouse model with reduced CD8^+^ T cell numbers [515], and the adoptive transfer of CD8^+^ T cells from p210 peptide of ApoB100-immunized mice slows plaque formation in recipients while expanding CD8 T cells [516], suggesting an antiatherogenic role for at least some CD8^+^ T cells. Indeed, atherosclerosis can be reduced by the cytolytic action of CD8^+^ T-cells on proatherogenic DCs [327] and follicular Th cells [517]. Moreover, the plaque microenvironment might potentiate a protective action of CD8 T cells by inducing expression of the ectonucleotidase CD39, which mediates decreased IFN-γ and TNF-α production in CD8 T cells [518].

Other studies support a proatherogenic role of CD8 T cells, as suggested by the increase in CD8^+^ T cell number as the lesion progresses toward more advanced stages [519], and the increased production of IFN-γ by CD28^+^CD8^+^ T cells in major artery-draining lymph nodes in hypercholesterolemic ApoE^−/−^ mice [520]. Furthermore, CD8 T cell stimulation by the injection of an anti-CD137 antibody increases inflammation and plaque development [521], and CD8 T cell depletion in LDLR^−/−^ mice reduces plaque formation by impeding monocyte hematopoiesis due to the decrease in systemic CD8 T-cell dependent IFN-γ [522]. CD8^+^ T cells have been reported to promote atherosclerosis through the induction of apoptosis in macrophages, endothelial cells, and smooth muscle cells, leading to necrotic core formation and increased inflammation [522]. A proatherogenic role is further suggested by the ability of CD8^+^ T cells to promote monocyte recruitment and augment lesion instability [513]. Overall, these studies, thus, suggest that different CD8 T cells subsets may play opposing roles in atherosclerosis [513].

### 5.3. B-Cells

B-cells play critical roles in innate and adaptive immunity through their capacity to produce antibodies and secrete cytokines. B cells are characterized by the expression of membrane-bound immunoglobulins, known as B cell receptor (BCRs), with exclusive epitope-binding sites. BCRs, each one unique to a single B cell clone, bind antigens and are generated by the recombination of variable (V), diversity (D), and joining (J) genes by the product of recombinase activation gene (RAG) [523,524]. The mature BCR and the subsequently produced antibodies are composed of duplicated copies of the recombined heavy and light immunoglobulin polypeptides. The BCR forms a complex with several other membrane proteins, including the B cell receptor CD22, receptor-type tyrosine protein phosphatase C (PTPRC, also known as B220 and CD45), and B-lymphocyte antigen CD19. BCR stimulation induces NF-κB, PI3K, and MAPK signaling, which play essential roles in B cell development, differentiation, and activation [525]. After activation, B cells proliferate and differentiate toward a plasma cell phenotype [525]. Differences in the Fc region define five major types of antibody: IgA, IgD, IgE, IgG, and IgM. Some of these can be divided into different subtypes [525].

B cells can be subclassified as B1 and B2 cells [526,527]. B1 cells derive from fetal hematopoietic stem cells and produce natural antibodies independently of Th signals, without infection or immunization [528,529]. B1 cells circulate systemically and also guard mucosal surfaces, where they generate immediate protection via antigen capture and opsonization of entering bacteria [530]. Like plasma cells, antibody-secreting B1 cells reside in the spleen and bone marrow in large numbers [530]. In mice, B1 cells are further classified as CD5-expressing B1a cells and B1b cells [531]. B1a cells produce spontaneous T cell-independent, long-lasting, unmutated IgM [532], whereas B1b cells produce IgA [533,534].

B2 cells derive from bone-marrow precursors, which, after several maturation steps in the bone marrow and the secondary lymphoid organs, differentiate into marginal zone (MZ) B cells and follicular (FO) B cells [526,527]. MZ B cells are found in the splenic marginal sinus and respond to blood-borne pathogens in a T cell-independent manner by differentiating toward antibody-secreting plasma cells [535]. FO B cells are found in the periphery and are activated by antigens and Th cells before undergoing the germinal center reactions (class-switch recombination and somatic hypermutation) to generate switched Igs and BCRs that more precisely react with the target antigen. At the germinal center, with the help of follicular DCs and follicular helper T cells, FO B cells undergo an affinity maturation and selection procedure for antigens and then give rise to antibody-precise, antibody-generating, long-lived plasma cells or memory B cells [532,536]. These plasma cells and memory B cells govern antibody-mediated immune responses. B cells also regulate T cell and macrophage differentiation and modulate inflammatory responses through the secretion of specific cytokines [537,538,539]. Regulatory B cells (Bregs) play immunosuppressive roles through the release of IL-10, TGFβ, and IL-35 [479,540,541,542].

B cells can play protective and pathogenic roles in atherosclerosis. An atheroprotective role is supported by the increased atherosclerosis in ApoE^−/−^ mice after splenectomy, which reduces B cells’ numbers; this effect is attenuated by the adoptive transfer of splenic B cells [543]. Moreover, atherosclerosis is increased after the reconstitution of bone marrow-depleted LDLR^−/−^ mice with bone marrow from B cell-deficient mice (µMT^−/−^ mice) [544], and this effect is also reversed by the adoptive transfer of splenic B cells [545]. Immunization studies have confirmed the antiatherogenic role of B cell-derived antibodies [532], and atherosclerosis is reduced upon immunization with malondialdehyde modified LDL (MDA-LDL) [546] or other oxLDLs [547].

A proatherogenic action of B cells is revealed in ApoE^−/−^ and LDLR^−/−^ mice upon depletion of B2 cells with antibodies to CD20, the receptor for the B-cell survival regulator B cell activating factor receptor (BAFFR) or by genetic disruption of BAFFR [548,549,550,551,552,553]

The different B cell subclasses appear to make distinct contributions to the B cell-mediated modulation of atherosclerosis [42,532]. B1a cells produce natural IgM antibodies against oxLDL and antigens derived from apoptotic cells, blocking macrophage oxLDL uptake and foam-cell generation and stimulating apoptotic cell removal from atherosclerotic plaques [554,555]. Experiments in splenectomized ApoE^−/−^ mice show that this atheroprotective effect is mediated by TLR4 and MYD88 [556]. The role of B1b cells in atherosclerosis is not fully understood; however, adoptive transfer of B1b cells to Rag1^−/−^/ApoE^−/−^ mice reduces atherosclerosis [557].

Atheroprotective B1 responses appear to depend on the control of the adaptive germinal center by B Cell Fcγ Receptor IIb [558]. The studies cited above suggest a proatherogenic action of B2 cells [548,549,550,551,552]. Bregs exert immunosuppressive functions by secreting the cytokines IL-10 and TGFβ [559,560,561], and IL-10 disruption promotes inflammatory cell infiltration and cytokine generation and enhances atherosclerosis in mice [471,543]. An atheroprotective role of Bregs is supported by increased IL-10 production and Treg induction after the transfer of CTB-p210-pulsed Bregs [562] or CD21hiCD23hiCD24hi Bregs [563]. However, another study found no protective effect of Bregs [548]. Further experiments are, therefore, needed to clarify this role.

## 6. Discussion

Emerging evidence indicates that LDLs stimulate both innate and adaptive immunity in atherosclerosis. Atherosclerosis is aggravated by proinflammatory responses mediated by macrophages, Th1 cells, and B2 cells. The action of proinflammatory immune-system components is reduced by regulatory cells, such as Tregs, Bregs, M2 macrophages, and tolerogenic DCs. Regulatory-cell–mediated approaches, thus, have great potential for future therapies. However, recent findings indicate that these immunosuppressive responses can become dysfunctional due to microenvironmental factors that convert protective functions into proatherogenic responses. Several immune cells, including CD8 T cells, B1 cells, Th2 cells, Th17 cells, and some DC subsets appear to play both a protective and a proatherogenic role in the onset and progression of atherosclerosis depending on other factors, or their true function remains unresolved. Further research is, therefore, need to elucidate the specific function of these cells in human atherosclerotic disease and develop therapeutic strategies that stimulate their protective actions.

Some studies in mice indicate that the inhibition of inflammatory processes in advanced atherosclerotic lesions allows atheroprotective immune responses to promote plaque regression. In humans, the CANTOS trial of the anti-IL-1β blocking antibody canakinumab in patients with a history of myocardial infarction and elevated serum hsCRP (high-sensitivity C-reactive protein) demonstrates that inflammation is a direct cause of the onset and development of atherosclerosis [564]. This clinical trial also highlights the importance of inflammation as a target for future treatments to reduce atherosclerosis. However, low dose methotrexate (MTX) treatment—included in the CIRT study [565] and aimed to reduce inflammation and cardiovascular risk in patients with previous myocardial infarction or multivessel coronary disease who have indirect evidence of inflammatory risk, by additionally having either type 2 diabetes or the metabolic syndrome [566]—fails to reduce cardiovascular events. Contrasting with anti-IL-1β blocking antibody canakinumab treatment, MTX did not result in lower IL-1β, IL-6, or hsCRP levels than placebo. This may due to the fact that MTX function and molecular targets are different from the canakinumab, affecting less relevant pathogenic pathways in atherosclerosis. Moreover, CANTOS and CIRT trials also differ in the population of study [567]. Nevertheless, it is important to weigh the potential dangers of this type of anti-inflammatory therapy; risks include aggravating or potentiating undesired effects, such as infection and cancer. In the CANTOS trial, canakinumab increased the incidence of fatal infection and sepsis [564] and in the CIRT study, a slight increase in skin tumors was observed over time in the MTX treated population [565]. Further studies are needed to determine the importance for the essential inflammatory responses of many potential therapeutic targets in the prevention and treatment of atherosclerosis.
